# Different methodological approaches to the assessment of *in vivo *efficacy of three artemisinin-based combination antimalarial treatments for the treatment of uncomplicated falciparum malaria in African children

**DOI:** 10.1186/1475-2875-7-154

**Published:** 2008-08-09

**Authors:** Elizabeth A Ashley, Loretxu Pinoges, Eleanor Turyakira, Grant Dorsey, Francesco Checchi, Hasifa Bukirwa, Ingrid van den Broek, Issaka Zongo, Pedro Pablo Palma Urruta, Michel van Herp, Suna Balkan, Walter R Taylor, Piero Olliaro, Jean-Paul Guthmann

**Affiliations:** 1 Epicentre, 8 rue Saint Sabin, 75011, Paris, France; 2 Department of Medicine, San Francisco General Hospital, University of California, San Francisco, California, USA; 3 London School of Hygiene and Tropical Medicine, Keppel St, London, UK; 4 Uganda Malaria Surveillance Project, Kampala, Uganda; 5 Médecins sans Frontières, Amsterdam, The Netherlands; 6 Center for Infectious Disease Control, National Institute for Public Health and the Environment, PO Box 1, 3720 BA Bilthoven, The Netherlands; 7 Institut de Recherche en Sciences de la Santé, Bobo-Dioulasso, Burkina Faso; 8 Médecins sans Frontières, Barcelona, Spain; 9 Médecins sans Frontières, Brussels, Belgium; 10 Médecins sans Frontières, Paris, France; 11 Oxford University Clinical Research Unit, National Institute for Infectious and Tropical Diseases, Hanoi, Vietnam; 12 Travel and Migration Medicine Unit, Geneva University Hospital, Rue Micheli-du-Crest 24, 1211 Geneva, 14, Switzerland; 13 UNICEF/UNDP/World Bank/WHO Special Programme on Research and Training in Tropical Diseases, WHO, Geneva, Switzerland; 14 Centre for Tropical Medicine and Vaccinology, Nuffield Department of Medicine, University of Oxford, Churchill Hospital, Oxford, OX3 7LJ, UK; 15 Unité Maladies à Prévention Vaccinale, Département des Maladies Infectieuses, Institut de Veille Sanitaire, 12, rue du Val d'Osne, 94415, Saint-Maurice, cedex, France

## Abstract

**Background:**

Use of different methods for assessing the efficacy of artemisinin-based combination antimalarial treatments (ACTs) will result in different estimates being reported, with implications for changes in treatment policy.

**Methods:**

Data from different *in vivo *studies of ACT treatment of uncomplicated falciparum malaria were combined in a single database. Efficacy at day 28 corrected by PCR genotyping was estimated using four methods. In the first two methods, failure rates were calculated as proportions with either (1a) reinfections excluded from the analysis (standard WHO per-protocol analysis) or (1b) reinfections considered as treatment successes. In the second two methods, failure rates were estimated using the Kaplan-Meier product limit formula using either (2a) WHO (2001) definitions of failure, or (2b) failure defined using parasitological criteria only.

**Results:**

Data analysed represented 2926 patients from 17 studies in nine African countries. Three ACTs were studied: artesunate-amodiaquine (AS+AQ, N = 1702), artesunate-sulphadoxine-pyrimethamine (AS+SP, N = 706) and artemether-lumefantrine (AL, N = 518).

Using method (1a), the day 28 failure rates ranged from 0% to 39.3% for AS+AQ treatment, from 1.0% to 33.3% for AS+SP treatment and from 0% to 3.3% for AL treatment. The median [range] difference in point estimates between method 1a (reference) and the others were: (i) method 1b = 1.3% [0 to24.8], (ii) method 2a = 1.1% [0 to21.5], and (iii) method 2b = 0% [-38 to19.3].

The standard per-protocol method (1a) tended to overestimate the risk of failure when compared to alternative methods using the same endpoint definitions (methods 1b and 2a). It either overestimated or underestimated the risk when endpoints based on parasitological rather than clinical criteria were applied. The standard method was also associated with a 34% reduction in the number of patients evaluated compared to the number of patients enrolled. Only 2% of the sample size was lost when failures were classified on the first day of parasite recurrence and survival analytical methods were used.

**Conclusion:**

The primary purpose of an *in vivo *study should be to provide a precise estimate of the risk of antimalarial treatment failure due to drug resistance. Use of survival analysis is the most appropriate way to estimate failure rates with parasitological recurrence classified as treatment failure on the day it occurs.

## Background

**Figure 1:**
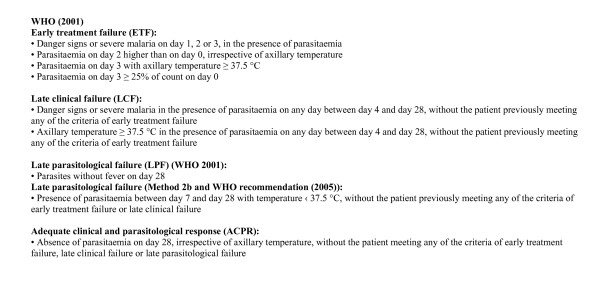
**Classification of efficacy endpoints (WHO, 2001)**.

Studies of antimalarial drug efficacy remain the primary source of evidence for treatment policy decisions. How these studies should be conducted and interpreted is still a subject for debate. Contentious methodological issues include length of follow-up after treatment, whether to use clinical or parasitological outcomes and which analytical methods are the most appropriate [1-4]. Study designs and data analysis vary; this makes combining and comparing data difficult. The World Health Organization (WHO) has made considerable efforts to standardize methods for the assessment of antimalarial drug efficacy over the last 40 years but these have changed several times, in line with the prevailing opinion at the time. The first *in vivo *test was developed in 1965 and was designed for the assessment of chloroquine efficacy against falciparum malaria using strictly defined parasitological end points. The protocol was revised in 1967 and 1972, with patients followed up for 28 days and kept in a mosquito-free environment to prevent re-infection. Subsequent modifications to the protocol in 1996 permitted a shorter length of follow-up of 14 days in areas of high transmission, where the main endpoint changed from parasitological clearance without subsequent recrudescence to adequate clinical response, i.e. a patient in whom parasites reappeared without symptoms was still regarded as 'cured'. In 2001, the length of follow-up was increased to 28 days in areas of intense transmission, if PCR genotyping was available, and the concept of late treatment failure incorporating clinical or parasitological failures was introduced. Asymptomatic patients with parasitological failure were followed from the day of reappearance to the last day of follow-up when they were treated if parasites were still present [5-7]. In a more recent document, 'Susceptibility of *Plasmodium falciparum *to antimalarial drugs', an update of the therapeutic efficacy test and modification of the protocol have been proposed with late parasitological failure defined as "presence of parasitaemia between day 7 and day 28 with temperature ‹37.5°C, without the patient previously meeting any of the criteria of early treatment failure or late clinical failure" [8]. Definitions of endpoints are summarized in Figure 1.

### Analysis of efficacy data

The recommended method for analysis of efficacy data changed in the 2001 WHO protocol to survival analysis. In the 2001 report, the life-table method is mentioned and, in the more detailed 2003 guidelines, there is an annex enabling users to calculate Kaplan-Meier survival curves by hand. In order for data to be comparable with earlier results, it was advised that "data [also] be analysed using the traditional 'per-protocol' approach", i.e. failure rates expressed as proportions with exclusion of patients lost from the study for any reason other than genotyping-confirmed recrudescence before the last day of follow-up. An Excel™ programme was developed to enable users to get their results analysed with per-protocol and Kaplan-Meier methods. In practice, survival analysis has not been used widely, but consensus appears to be growing that it is the method of choice [1,4,9].

### Polymerase chain reaction genotyping

PCR genotyping allows the reappearance of parasites during follow-up to be classified as a new or recrudescent infection. However the technique is not without its limitations. Methods are not standardized with different laboratories using different numbers of genotypic markers and different methods of interpretation [10]. Distinguishing a new from a recrudescent infection is particularly challenging in areas of high transmission where multiple genotype infections are common [11]. Identification of an identical genotype during follow-up is not absolute proof of recrudescence just as identification of a different genotype could indicate recrudescence of a previously undetected minority genotype. The WHO has recommended since 2003 that for data analysis "re-infections (as well as PCR unclassifiable results) be classified as involuntary withdrawals" and, therefore, excluded from the analysis.

### Implications of using different analytical methods

The differences which may result from use of different methods of analysis are important because results of *in vivo *studies using the WHO protocol are used to guide changes in treatment policy. Current recommendations (WHO 2006) are that a new antimalarial drug combination should be 95% efficacious when deployed and change should be considered when efficacy falls to 90% [12].

### Data sources

Since 2002, Epicentre/Médecins sans Frontières and the Department of Medicine, San Francisco General Hospital, University of California, San Francisco (UCSF), in partnership with Ministries of Health or Universities in a number of African countries have conducted studies of the efficacy of artemisinin-based combination regimens. All studies underwent ethical review and were approved by the relevant national authorities [13-26].

### Study objectives

These data were combined for this analysis with the following objectives-

(a) to examine the effect of using different definitions of treatment failure and methods of analysis on estimates of PCR-corrected failure rates.

(b) to describe the utility of PCR genotyping to classify parasite recurrences in *in vivo *studies.

## Methods

### Criteria for inclusion or exclusion of data

Studies were included if the treatment given was a supervised therapeutic regimen of three days of an artemisinin derivative combined with another antimalarial, and complied with the WHO 2001 guidelines, with follow-up for at least 28 days and PCR genotyping to distinguish re-infections from recrudescences.

### Overview of design of studies and methods

In brief, children aged 6–59 months were eligible for enrolment if they had *P. falciparum *mono-infection (density threshold at entry varying with study site from a minimum of 500 to 2,000/μL up to a maximum of 100,000 or 200,000/μL), met none of the criteria for severe malaria, no reported hypersensitivity to the study drug, no serious concomitant febrile illness and consent given by accompanying caregivers. Subjects were re-assessed clinically and parasitologically on days 1, 2, 3, 7, 14, 21 and 28, or any day in-between in case of illness. In the studies conducted by Epicentre/MSF, when asymptomatic parasitaemia occurred after day 3, children were monitored closely and administered a rescue antimalarial (quinine), if fever was measured in the clinic, or if they were still parasitaemic without symptoms on day 28. In the UCSF collaborative studies cases of recurrent parasitaemia with a history of fever were treated.

### PCR genotyping methods

PCR genotyping was used to classify parasite recurrences occurring after day 14 of follow up. All recurrences occurring on or before day 14 were considered to be recrudescent infections. Genotyping was done at different laboratories that used different extraction methods, primers and reaction conditions (Table 1). For the Epicentre/MSF studies, cases in which the post-treatment sample featured all or some of the pre-treatment alleles were considered recrudescent infections; cases in which pre- and post-treatment genotypes were entirely different were considered as new infections. This approach was modified to classify recurrences of parasitaemia in the UCSF collaborative studies as follows:

**Table 1:** Laboratories performing PCR genotyping with assessment of PCR use-effectiveness in 17 *in vivo *studies of antimalarial drug efficacy

**Year**	**Country** ** & Site**	**Laboratory**	**Method used**	**N**	**Unresolved** **by PCR** n	**Resolved** **by PCR** n	**PCR Use-** **effectiveness** ** [95%CI]**
							
				**recurr.** **after** ** day14**			
2002	Zambia, Maheba	Rouen France	*msp2*	43	3	40	**93.0**
							**[80.9–98.5]**
2003	Angola, Kuito	IMT Antwerp	*msp1+msp2*	8	0	8	**100.0**
							**[63.1–100.0]**
2003	DRC, Boende	IMT Antwerp	*msp1+msp2 +glurp*	78	10	68	**87.2**
							**[77.7–93.7]**
2003	S Sudan, Nuba	Wellcome Trust/Medical College of Malawi	*msp1+msp2*	54	15	39	**72.2**
							**[58.3–83.5]**
2003	Uganda, Amudat	Epicentre Mbarara	*msp1+msp2 +glurp*	73	4	69	**94.5**
							**[86.6–98.5]**
2003	Uganda, Jinja	UCSF	*msp2*	27	2	25	**92.6**
							**[75.7–99.1]**
2003	Uganda, Kampala	UCSF	*msp2*	14	0	14	**100.0**
							[76.8–100.0]*
2004	Angola, Caala	Mbarara University	*msp1+msp2 +glurp*	6	2	4	**66.7**
							**[22.3–95.7]**
2004	Congo, Kindamba	SMRU	*msp1+msp2 +glurp*	60	24	36	**60.0**
							**[46.5–72.4]**
2004	DRC, Kabalo	Epicentre Mbarara	*msp1+msp2 +glurp*	10	1	9	**90.0**
							**[55.5–99.7]**
2004	Guinea, Dabola	Epicentre Mbarara	*msp1+msp2 +glurp*	14	1	13	**92.9**
							**[66.1–99.8]**
2004	Sierra Leone, Kailahun	Paris, Avicenne	*msp1+msp2*	65	1	64	**98.5**
							**[91.7–100.0]**
2004	Uganda, Apac	UCSF	*msp2*	76	3	73	**96.0**
							**[88.9–99.2]**
2004	Uganda, Arua	UCSF	*msp2*	61	4	57	**93.4**
							**[84.1–98.2]**
2004	Uganda, Tororo	UCSF	*msp2*	120	3	117	**97.5**
							**[92.9–99.5]**
2005	Burkina Faso, Bobo-Dioulasso	UCSF	*msp1 +msp2 *+ 4 microsatellites	24	0	24	**100**
							[85.7–100]*
2005	Uganda, Tororo	UCSF	*msp1+msp2*	209	4	205	**98.1**
							**[95.2–99.5]**

	**TOTAL**			942	77	865	

	**Weighted average [CI 95]**						**91.8 [89.9–93.4]**

	**Median [range]**						**93.4 [60–100]**

Use-effectiveness = number of children with parasite recurrence resolved by PCR/total number of children with recurrences after day 14.

* One sided 97.5% CIs have been calculated

IMT Institut de Médecine Tropicale Antwerp

KEMRI Kenya Medical Research Institute

SMRU Shoklo Malaria Research Unit, Thailand

UCSF University of California, San Francisco

*msp1 *merozoite surface protein 1

*msp2 *merozoite surface protein 2

*glurp *glutamate rich protein

(i) PCR outcomes defined as "recrudescence + re-infection" were reclassified as recrudescence if more the half the alleles on the day of failure were present on day 0 or as new infections for the remainder (Kampala, Uganda).

(ii) PCR outcomes defined as "recrudescence + re-infection" were classified as new infections (Apac, Arua, Tororo 2004, Jinja, Uganda).

(iii) PCR outcomes defined as "recrudescence + re-infection" were classified as new infections. MSP1 was also done for patients defined as "recrudescence" by MSP2. The final classification of recrudescence required that all of the alleles on the day of failure had to be present on day 0 for both MSP2 and MSP1 (Tororo 2005, Uganda).

(iv) MSP2, MSP1 and four microsatellites were used for all LCF and LPFs. To be classified as a recrudescence there had to be at least one allele present on the day of failure which was present on day 0 for all six loci (Burkina Faso 2005).

### Data analysis

Databases were imported into Stata 9.0 (Stata Corp., Texas, USA) and the variables of interest selected which were recoded according to a pre-determined list. Individual databases were verified against existing study reports if available. Discrepancies were resolved by consultation with the study principal investigators. Individual databases were merged into a single database for analysis.

### Evaluation of PCR genotyping methods

PCR genotyping results for children with a parasite recurrence after day 14 were classified as either resolved by PCR (recrudescence or re-infection), or unresolved by PCR, and the reason recorded (missing sample, failure to extract DNA, indeterminate result).

The use-effectiveness of the PCR genotyping method was assessed, defined as the number of children with parasite recurrence resolved by PCR divided by the total number of children with recurrences after day 14 [27].

### Efficacy analyses

**Figure 2:**
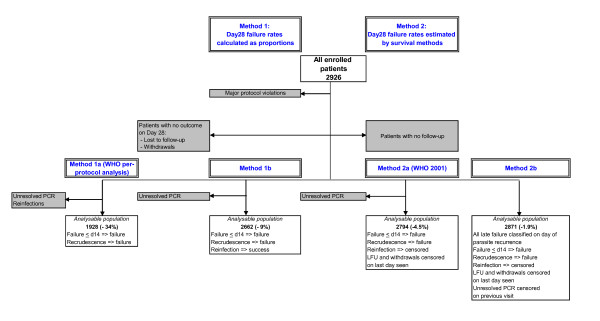
**Summary of different analyses**. Notes: Shaded boxes represent patients excluded from the analyses. Analysable populations – Figures in parentheses represent the % patients excluded from the enrolled patient population using each method of analysis.

Four different analyses were done. Figure 2 presents the population selected and the methods used for each analysis. The populations differ depending on both the definition of failure and the analytical method used. Efficacy results are presented as point estimates for each drug in each study without making comparisons between treatment arms in the same study. Patients wrongly randomized, who did not meet study inclusion criteria were excluded from all analyses. Withdrawals for other reasons were dealt with in different ways depending on the analytical method used as explained below. Failure rates were calculated as proportions (methods 1a and 1b) or estimated using survival analytical methods (methods 2a and 2b) as follows-

(i) Day 28 failure rates calculated as proportions using a per-protocol dataset-

Patients with no outcome on day 28 (losses to follow up and withdrawals) and patients with recurrence unresolved by PCR were excluded from these analyses. New infections during the follow-up were treated in 2 ways:

• *Method 1a*: individuals with new infections excluded from the analysis. This is the standard per-protocol analysis reported by most investigators following the WHO protocol and for convenience will be referred to as the "WHO per protocol method" to indicate failure rates calculated as proportions and endpoints defined as in the WHO (2001) protocol.

• *Method 1b*: all PCR-confirmed new infections re-classified as successes (ACPR).

(ii) Day 28 failure rates estimated by the Kaplan-Meier product limit formula (survival analysis) using the dataset of correctly randomized patients -

Losses to follow-up and withdrawals were included in this analysis and were censored on the last day seen. This analysis was done using two different definitions of failure -

• *Method 2a*: The definition of failure included all early treatment failures plus late treatment failures (LCFs or LPFs classified on day 28 as defined by WHO (2001)). Patients classified as LCF with a reinfection were censored. Patients with unresolved PCR results were excluded (n = 74).

• *Method 2b*: The definition of failure included all early treatment failures plus late treatment failures (LTF), which were defined on day 7 in patients whose initial parasitaemia had not cleared, or on the first day of any parasite recurrence during follow-up (including day 7 or earlier), irrespective of symptoms. For this analysis, patients with reinfections were censored on the day of parasite reappearance and those with indeterminate or missing genotyping results were censored on the last visit with a negative malaria smear result documented [4]. The date the PCR was taken was not the date of parasite recurrence in all cases and genotyping results were missing for a small number of participants (N = 16) because their recurrent parasites had resolved without further treatment by day 28, and thus using the WHO (2001) classification they had been classified as ACPRs at the time the study was done. These patients were censored at the time of the previous visit when the malaria smear was negative.

### Effect of method of assessment of sample size evaluated

The numbers of patients included in the four efficacy analyses were compared and the percentage of patients excluded from the enrolled patient population calculated for each method (Figure 2).

## Results

Out of 21 studies in which 4,684 patients were in an ACT treatment arm, 17 were included in the analysis representing a total of 2926 children treated with an ACT. Reasons for excluding studies or patients were major deviations from the WHO protocol (1257 patients), patients greater than five years of age enrolled (335), or unacceptable laboratory quality control results (166 patients in one study). Most studies (12/17) were in areas described as hyperendemic for malaria.

The most commonly studied ACT was artesunate-amodiaquine (AS+AQ) (15 sites, 1,702 children), followed by artesunate-sulphadoxine-pyrimethamine (AS+SP) (eight sites, 706 children) and artemether-lumefantrine (AL) (four sites, 518 children).

### Description of treatment outcomes according to the WHO 2001 classification

There were four early treatment failures (ETF), 635 late clinical failures (LCF) and 376 late parasitological failures (LPF). Only 2% patients were lost to follow-up. There were 127 (4%) withdrawals in all studies. The reasons for withdrawal were repeated vomiting (20/127), taking another drug with antimalarial activity (23/127), concomitant disease (23/127), withdrawal of consent (7/127), other protocol violation (48/127), death (1/127) and unknown (5/127).

### Results of efficacy analyses

Efficacy results are summarized in Tables 2, 3 and 4. Using method 1a (per protocol) day 28 failure rates ranged from 0% to 39.3% for AS+AQ treatment (Table 2), from 0% to 3.3% for AL treatment (Table 3) and from 1.0% to 33.3% for AS+SP treatment (Table 4). Failure rates exceeded 10% at 7/15 sites for AS+AQ, 0/4 sites for AL and 4/8 sites for AS+SP. The upper 95% CI exceeded 10% at 11 sites for AS+AQ, no sites for AL and 6 sites for AS+SP.

**Table 2:** Day 28 failure rates estimated by four methods – AS+AQ treatment

		**Day 28 failure rates** % [CI95%]			
		
**Year**	**Country &** ** Site**	Method 1a (per protocol)	Method 1b	Method 2a (WHO 2001)	Method 2b
2003	Angola, Kuito	1.3	1.2	1.2	1.2
		[0–6.8]	[0–6.4]	[0.2–8.3]	[0.2–8.1]
2003	DRC, Boende	18.3	14.1	15.3	56.3
		[9.5–30.4]	[7.2–23.8]	[8.7–25.9]	[45.8–67.4]
2003	S Sudan, Nuba	7.3	5.6	6.2	8.4
		[2.0–17.6]	[1.5–12.6]	[2.4–15.7]	[3.9–18.0]
2003	Uganda, Amudat	22.6	8.8	10.4	21.4
		[9.6–41.1]	[3.6–17.2]	[4.9–21.1]	[14.0–31.8]
2003	Uganda, Jinja	7.9	6.5	7.0	12.2
		[3.2–15.7]	[2.7–13.0]	[3.4–14.1]	[7.2–20.1]
2003	Uganda, Kampala	4.9	4.1	4.3	4.3
		[1.0–13.7]	[0.9–11.5]	[1.4–12.8]	[1.4–12.9]
2004	Angola, Caala	0	0	0	1.5
		[0–6.0]*	[0–5.7]*	[0–5.8]*	[0.2–10.4]
2004	Congo, Kindamba	6.0	4.8	4.8	6.4
		[1.6–14.6]	[1.3–11.9]	[1.8–12.2]	[2.9–13.7]
2004	DRC, Kabalo	0	0	0	4.4
		[0–8.4]*	[0–7.9]*	[0–7.9]*	[1.1–16.3]
2004	Guinea, Dabola	1.0	0.9	0.9	0.9
		[0–5.3]	[0–5.1]	[0.1–6.5]	[0.1–6.5]
2004	S Leone, Kailahun	27.0	15.4	19.0	20.5
		[16.6–39.6]	[9.3–23.6]	[12.3–28.8]	[13.5–30.5]
2004	Uganda, Apac	24.5	15.0	15.7	15.9
		[16.2–34.4]	[9.8–21.7]	[10.7–22.7]	[10.8–23.1]
2004	Uganda, Arua	28.7	19.9	20.0	20.0
		[20.4–38.2]	[13.9–27.0]	[14.5–27.2]	[14.6–27.0]
2004	Uganda, Tororo	39.3	14.5	17.8	20.0
		[27.1–52.7]	[9.5–20.9]	[12.1–25.8]	[13.6–28.9]
2005	Uganda, Tororo	13.9	5.5	5.4	5.9
		[6.9–24.1]	[2.6–9.8]	[2.9–9.8]	[3.3–10.4]

*one-sided 97.5% CIs

**Table 3:** Day 28 failure rates estimated by four methods – AL treatment

		**Day 28 failure rates** % [CI95%]			
		
		Method 1a (per protocol)	Method 1b	Method 2a (WHO 2001)	Method 2b
2004	Angola, Caala	0	0	0	0
		[0–6.1]*	[0–6.0]*		
2004	Congo, Kindamba	1.2	1.1	1.1	1.0
		[0–6.2]	[0–6.0]	[0.2–7.3]	[0.1–6.8]
2005	Burkina Faso, Bobo-Dioulasso	3.2	2.8	2.8	2.8
		[0.9–8.1]	[0.8–6.9]	[1.1–7.3]	[1.1–7.3]
2005	Uganda, Tororo	3.3	1.6	1.8	1.8
		[0.7–9.3]	[0.3–4.7]	[0.6–5.4]	[0.6–5.7]

*one-sided 97.5% Cis

**Table 4:** Day 28 failure rates estimated by four methods – AS+SP treatment

		**Day 28 failure rates**			
		
**Year**	**Country &** ** Site**	Method 1a (per protocol)	Method 1b	Method 2a (WHO 2001)	Method 2b
2002	Zambia, Maheba	21.7	13.2	15.4	29.8
		[10.9–36.4]	[6.5–22.9]	[8.6–26.8]	[20.5–42.1]
2003	Angola, Kuito	1.2	1.2	1.1	2.3
		[0–6.6]	[0–6.4]	[0.2–7.8]	[0.6–8.8]
2003	DRC, Boende	33.3	25.3	27.3	59.5
		[21.9–46.3]	[16.4–36.0]	[18.7–38.9]	[48.8–70.5]
2003	S Sudan, Nuba	10.3	8.3	8.6	10.5
		[3.9–21.2]	[3.1–17.3]	[4.0–18.2]	[5.4–19.9]
2003	Uganda, Amudat	7.9	6.8	6.8	8.0
		[2.6–17.5]	[2.2–15.1]	[2.9–15.5]	[3.7–16.9]
2004	Congo, Kindamba	11.1	10.0	10.1	11.0
		[4.9–20.7]	[4.4–18.8]	[5.2–19.2]	[5.9–20.2]
2004	DRC, Kabalo	2.3	2.0	2.0	11.8
		[0–12.3]	[0–10.8]	[0.3–13.4]	[5.5–24.4]
2004	Guinea, Dabola	1.0	0.9	0.9	2.8
		[0–5.5]	[0–5.2]	[0.1–6.5]	[0.9–8.5]

Using method 1a as the reference analytical method, the median [range] differences between these results and those obtained using method 1b were 1.3% [0–24.8], method 2a 1.1% [0–21.5] and method 2b 0% [-38–19.3].

Classifying patients as failures on day 7 if initial parasitaemia had not cleared, or on the first day of parasite recurrence, regardless of symptoms (method 2b) increased the number of treatment failures (uncorrected by genotyping) by 75, from 1015 to 1090 but it should be noted that the majority of these patients (50) were from one site (Boende).

### Effect of method of assessment on sample size evaluated

Figure 2 illustrates how the choice of definitions and methods for assessment of efficacy (corrected by PCR) affected the number of patients assessed in the final efficacy evaluation. The biggest reduction in sample size of 34% occurred using the per-protocol analysis (method 1a). The smallest reduction in sample size occurred when method 2b was applied.

### PCR findings

Recurrence of parasitaemia between days 14 and 28 occurred in 942 (32%) children. Of these, 77 (8.3%) patients could not be classified because: (i) 18 samples were missing, (ii) in 31 samples it was not possible to amplify any parasite DNA, (iii) DNA was amplified but no classification could be given (indeterminate) in 26 samples, and (iv) the reason was unknown for two samples.

A total of 865 recurrences were categorized following PCR genotyping with: (i) 118 (12.5%) classified as recrudescent, (ii) 734 (77.9%) as re-infections, and (iii) 13 (1.4%) as recrudescence + re-infection, regarded as recrudescent for the purposes of this analysis. The use-effectiveness was 91.8% (865/942) and ranged from 60.0% for Congo-Kindamba to 100.0% for Angola-Kuito and Uganda-Kampala and Bobo-Dioulasso, Burkina Faso (Table 1).

## Discussion

This analysis of pooled data has shown that failure rates can vary markedly depending on the analytical method used. The point estimates of failure calculated using the per-protocol analysis compared to methods 1b and 2a were the highest. This systematic overestimation is attributable to the effect of excluding patients with new infections from the analysis. Predictably, the magnitude of the difference from the estimate obtained using method 1b varied according to the number of patients re-infected during follow-up, hence intensity of transmission and the post-treatment prophylactic effect of the drug. In these studies, the effect of data loss due to patient loss to follow up (2%) and withdrawals (4%) was small over 28 days.

If the results of the survival analysis using the same definitions of failure (WHO 2001) are compared to the results of the per protocol analysis the median difference in the point estimates for failure at day 28 was small at 1.1% but ranged from 0 to 21.5%. Again, the difference was greater at the sites with the highest numbers of parasite recurrences, whether related to transmission intensity and more reinfections or a higher number of recrudescent infections. The precision of those estimates generated by the per protocol analysis was lower. Comparisons of precision cannot be made against methods 1b or 2b because the endpoint classifications were different.

The change in definition of failure from the WHO (2001) protocol to method 2b led to an increasing number of patients being classified as treatment failures (uncorrected by genotyping): 1090 compared to 1015. By classifying patients on day 7 or on the day of recurrence rather than by day 28 (method 2b vs. 2a), the results of the survival analyses changed little in most of the studies but markedly at certain sites where several patients who had been classified as ACPRs using the 2001 criteria were reclassified as late treatment failures using the 2005 definition, e.g. the Boende site in DRC where failure [95% CI] would have been estimated as 15.3% [8.7–25.9] when the study was done in 2003 and 56.3% [45.8–67.4] if it had been done 2 years later following the newer recommendations. This was because a relatively high proportion of patients who were still parasitaemic on days 7 (n = 29) and 14 (n = 21) but asymptomatic had cleared their parasites by day 28 without treatment.

The methods for assessing antimalarial drug efficacy continue to evolve. The quality of the data generated by an *in vivo *study depends on the study design and execution, the quality of the laboratory and the interpretation of the genotyping results. For the efficacy analysis, there is a growing consensus that the Kaplan-Meier survival analysis is the optimal method because it retains the maximal amount of data for analysis and avoids the inaccurate overestimation of failure rates that will occur when large numbers of patients are excluded from the analysis. This overestimation is likely to increase as a drug's efficacy declines with a consequent increase in the number of recrudescences and new infections. These results support expert opinion that survival analysis should become the standard way of analysing data from *in vivo *tests and should replace the traditional per-protocol method. The per-protocol method was associated with a 34% reduction in sample size, compared to a < 10% reduction for the other methods. However, it is true that while survival analysis maximizes the sample size, some of the patients will only have completed a trivial period of follow up. Regarding re-infections as treatment successes (method 1b) preserves the sample size but leads to an underestimation of the failure rate and is not recommended.

Efficacy of AS+AQ was below 90% at 7/15 sites and of AS+SP at 4/8 sites and the lower 95% CI was below this level at a further four and two sites, respectively. Efficacy of AL was high in the small number of patients studied. The reduced efficacy of AS+AQ and AS+SP could be explained by the use of the more slowly eliminated partner drug as monotherapy before and during the deployment of the combinations. An alternative explanation for these results is different methods of classification by PCR genotyping. The results reported here suggest that PCR genotyping performed well as a discriminatory test inasmuch as the majority of patients were classified, but the results are only as valid as the interpretation algorithms used to classify them. Differences in performance were observed between different laboratories, which did not appear to be related to the number of markers used. PCR outcomes defined as recrudescence + re-infection were classified as new infections by laboratories in certain studies but not in others as described in the methods. Current genotyping techniques, even using up to six markers, are not sufficiently discriminatory in areas of high transmission where multiple genotype infections are common [11]. Standardization of genotyping techniques and interpretation rules should reduce this inter-laboratory variation. There is evidence from genotypic studies that re-infections can occur before day 14 [28]. More work needs to be done in this area and the current guidelines, stating that only parasite recurrences occurring after this day should be genotyped, may need to be revised.

These results support the recommendation for a minimum follow-up period of 28 days in the assessment of efficacy of ACTs, with PCR-genotyping of all recurrences and the use of the Kaplan-Meier product-limit formula to estimate efficacy of treatment in all correctly randomized patients.

Accurate reporting of methods by investigators is essential, in particular definitions of endpoints and criteria for censoring all categories of patients who do not complete follow-up. It is recommended that the classification of treatment failure is based on parasitological outcomes only and that all parasitological recurrences be treated straightaway in view of the high likelihood that patients will go on to develop symptoms [29]. Further standardization of definitions and analytical methods will facilitate combining data in this critical period post ACT-deployment, allowing trends in efficacy to be monitored at a global level [9,30].

The WHO (2001) protocol was not designed to be a comparative study protocol, but rather a single-arm *in vivo *assessment to guide treatment policy. Such studies should be powered to estimate the effect size with adequate precision. However, if an efficacy assessment is being done because of a suspicion a drug is failing it makes sense to evaluate a potential replacement at the same time in an adequately powered, randomized, controlled trial. This will provide good evidence to guide national programmes in their drug selections.

Whether *in vivo *efficacy monitoring data should be used as the only determinant for policy change is debatable, if a choice of highly efficacious therapies becomes available. When comparing treatments of similar efficacy other factors determining programmatic effectiveness become more important to policy makers such as simplicity of the regimen, formulation, tolerability, adherence, cost and post-treatment prophylactic effect.

## Competing interests

The authors declare that they have no competing interests.

## Authors' contributions

JPG, PO and WRT conceived of the study, and participated in its design and coordination and helped to draft the manuscript. EAA participated in its design, analysed some of the data and wrote the first draft of the manuscript. LP and ET did the statistical analysis and helped to draft the manuscript. GD, HB, IZ, IvdB, PPP, MvH, FC and SB collected data and revised the manuscript. All authors read and approved the final manuscript.
